# Supplementary material for: DNA Methylation of Neurodevelopmental, Stress-Response, Reward, and Opioid Pathway Genes: Sex Differences in Infants with Neonatal Opioid Exposure
Source: medRxiv. 2026 Sep 29:2026.09.28.26364198. Preprint. [Version 1] doi: 10.64898/2026.09.28.26364198 (PMC13648809; doi:10.64898/2026.09.28.26364198)
Supplement: Supplementary file 1 [file NIHPP2026.09.28.26364198V1-supplement-1.pdf]

medRxiv preprint doi: https://doi.org/10.64898/2026.09.28.26364198; this version posted September 29, 2026. The copyright holder for this preprint (which was not certified by peer review) is the author/funder, who has granted medRxiv a license to display the preprint in perpetuity.

It is made available under a CC-BY 4.0 International license .

Supplementary

Table 1: Maternal and Infant Characteristics by Inclusion Status

	Overall N = 235	***Included N = 181***	***Excluded N = 54***	
	M (SD) or N (%)	M (SD) or N (%)	M (SD) or N (%)	***p*-value**
Maternal Characteristics				
Less than high school degree	57 (24%)	45 (25%)	12 (23%)	0.77
Adequate prenatal care	189 (82%)	146 (81%)	43 (84%)	0.75
No partner	107 (46%)	77 (43%)	30 (57%)	0.11
Hypertension	49 (21%)	39 (22%)	10 (19%)	0.80
Residential Treatment	47 (20%)	40 (22%)	7 (13%)	0.20
Inpatient detox during pregnancy	41 (19%)	34 (20%)	7 (15%)	0.60
Bipolar	52 (22%)	47 (26%)	5 (9%)	0.02*
PTSD	85 (37%)	66 (37%)	19 (36%)	1.00
Hepatitis C	73 (32%)	60 (35%)	13 (25%)	0.22
Infant Characteristics				
Sex (Male)	120 (51%)	89 (49%)	31 (57%)	0.36
Gestational age (weeks)	38.25 (1.71)	38.29 (1.74)	38.12 (1.65)	0.52
Birth weight (grams)	3015.9 (542.6)	3006.41 (551.08)	3047.85 (517.03)	0.61
Head circumference (cm)	33.8 (2.19)	33.61 (1.88)	34.27 (2.96)	0.14
Birth length (cm)	48.4 (3.24)	48.45 (2.99)	48.05 (4.03)	0.52
Social Services involvement	114 (49%)	85 (47%)	29 (55%)	0.42
Breastfeeding	138 (59%)	108 (60%)	30 (56%)	0.70
Maximum Finnegan Score	10.1 (3.01)	10.3 (3.01)	9.26 (2.94)	0.08

Note: Percentages are calculated among participants with available data for each variable; denominators may therefore vary across measures. *$p < 0.05$
